# The Hippo pathway: potential target for immunotherapy of crescentic glomerulonephritis

**DOI:** 10.3389/fimmu.2025.1672128

**Published:** 2025-09-11

**Authors:** Huang Kuang, Zhao Cui, Ming-hui Zhao, Xiao-yu Jia

**Affiliations:** ^1^ Renal Division, Peking University First Hospital, Beijing, China; ^2^ Institute of Nephrology, Peking University, Beijing, China; ^3^ Key Laboratory of Renal Disease, Ministry of Health of China, Beijing, China; ^4^ Key Laboratory of CKD Prevention and Treatment, Ministry of Education of China, Beijing, China

**Keywords:** crescentic glomerulonephritis, Hippo pathway, podocyte, parietal epithelial cell, cross-talk, immunotherapy

## Introduction

Crescentic glomerulonephritis (cGN) represents a severe form of glomerular injury with rapidly progressive glomerulonephritis and pathological diffuse glomerular crescents constitute its hallmark features. cGN often leads to end-stage kidney disease within a few months and thus requires lifelong dialysis. It is categorized into three main types based on pathogenesis and immunofluorescence patterns: anti-glomerular basement membrane (GBM) disease, antineutrophil cytoplasmic antibody (ANCA)-associated vasculitis, and immune complex-mediated cGN. Although the etiology of the different types of cGN varies, they all ultimately lead to the formation of crescents composed predominantly of proliferative parietal epithelial cells (PECs). Anatomically, crescents exist between the podocytes-associated glomerular filtration barrier and quiescent PECs-formed Bowman’s capsule. Such a spatial correlation and same developmental origins of podocyte and PECs (1), rendering the hypothesis that cross-talk between podocytes and PECs may be a shared downstream mechanism involved in the pathogenesis of cGN. To this end, Turinsky et al. recently investigated a pivotal role of the podocyte-specific Hippo-YAP activation through cross-talk with PECs in the pathogenesis of cGN (2).

## Key signals in the Hippo pathway

**Figure 1:**
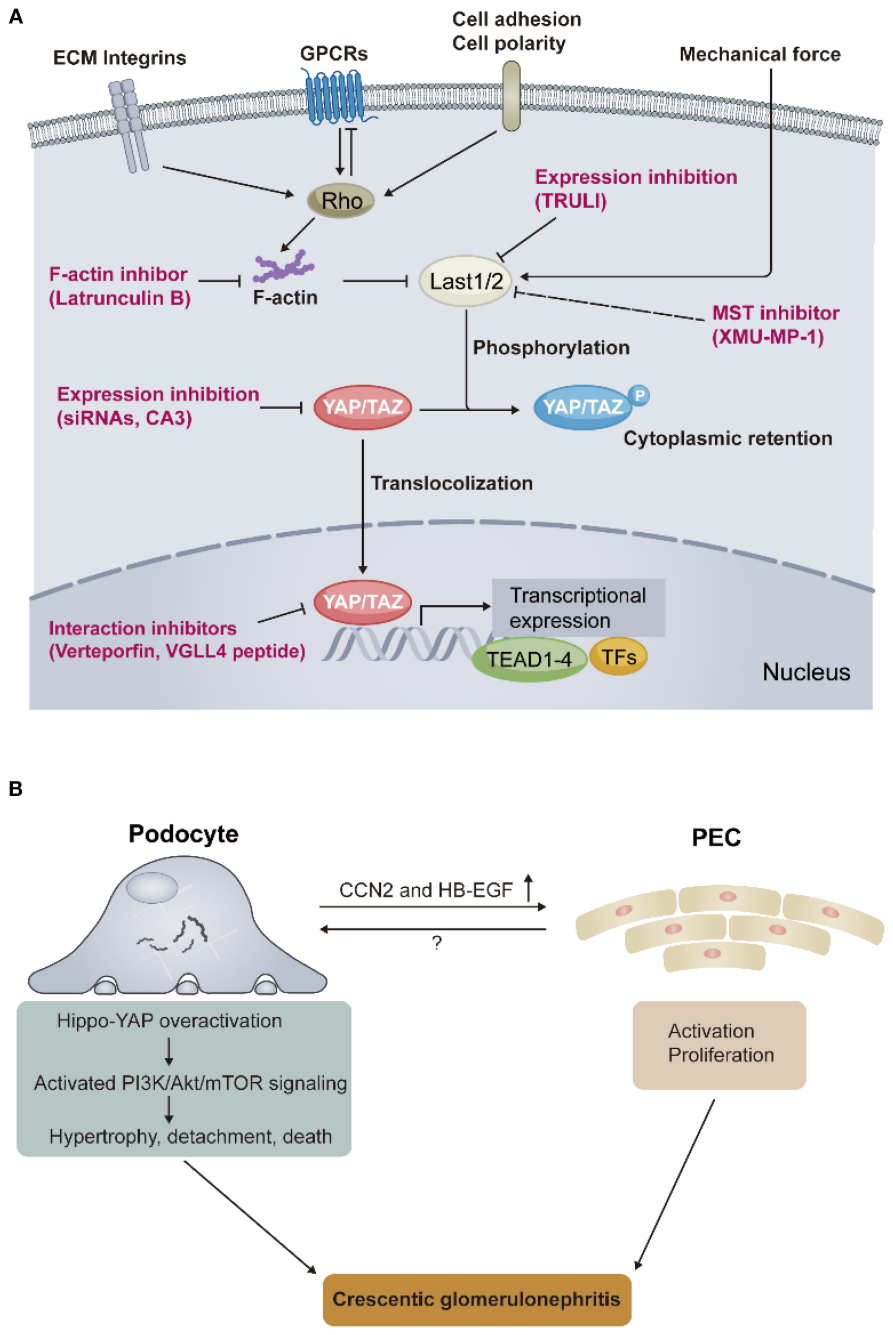
Core signals in the Hippo pathway and the role of Hippo-YAP overactivation in the pathogenesis of crescentic glomerulonephritis. **(A)** The Hippo pathway integrates multiple upstream signals to regulate YAP/TAZ activity, such as extracellular matrix (ECM) components integrins, G protein–coupled receptors (GPCRs), cell adhesion and polarity, and mechanical force. The dynamic localization of YAP/TAZ between the nucleus and the cytoplasm controls the transcriptional expression of downstream target genes. Red words depict the strategies for targeting YAP/TAZ activity. **(B)** Schematic representation of key findings regarding how YAP overactivation links podocyte injury and its cross-talk with partial epithelial cells (PECs), resulting in crescentic glomerulonephritis, found by Turinsky et al. Rho, Rho guanosine triphosphatases (GTPases); Last1/2, large tumor suppressor kinase 1/2; YAP, yes-associated protein; TAZ, WW-domain-containing transcription regulator 1; TEAD, transcriptional enhanced associated domain; TFs, transcriptional factors; VGLL4, vestigial-like family protein; siRNA, small interfering RNA; TRULI, CA3, and XMU-MP-1, small molecule inhibitors; PI3K/Akt/mTOR, phosphoinositide 3-kinase–protein kinase B–mechanistic target of rapamycin; CCN2, cellular communication network factor 2; HB-EGF, heparin-binding epidermal growth factor–like growth factor.

The Hippo pathway was first uncovered and defined in *Drosophila*, where its tumor-suppressive roles were identified due to mutations in genes encoding key effectors that lead to tissue overgrowth. It was further confirmed as a highly conserved signaling cascade that widely participates in various human cancers and other diseases, with essential functions in organ development, fibrosis, and regeneration. Transcriptional coactivators, yes-associated protein (YAP) and its paralog, PDZ binding motif (TAZ), as core effectors of the Hippo pathway, mediate the biological functions when localized in the nucleus, directing the activity of a range of transcription factors. The well-documented transcription factors regulated by YAP/TAZ are the TEAD family (TEAD1-TEAD4). In the Hippo pathway, YAP/TAZ are predominantly directly regulated by LATS1/2 through phosphorylation, which promotes cytoplasmic localization and thus inhibits YAP/TAZ transcriptional activity. **Figure 1A** depicts the key signals regulating YAP/TAZ activity in the Hippo pathway. Numerous upstream signals indirectly regulate YAP/TAZ activity, such as extracellular matrix (ECM), mechanical force, cell adhesion and polarity, and GPCRs (3).

## The Hippo pathway and kidney

In mammals, the Hippo pathway has been identified in participating in embryonic kidney development, kidney fibrosis, and the maintenance of podocyte homeostasis (4). Unlike quiescent PECs, which reside along the Bowman’s capsule of the outer glomerulus, podocytes maintain the capillary loop architecture within the glomerulus to establish the glomerular filtration barrier, making they continuously face physiological mechanical filtration force. This truth reasonably renders a hypothesis that podocytes are inclined to be regulated by the Hippo pathway when they encounter injury. More recently, the ECM component integrins α3β1 and αvβ5 on podocytes, which regulate YAP/TAZ activity, were confirmed crucial for the maintenance of normal podocyte function (5), indicating that cell adhesion-driven Hippo-YAP signaling activation is involved in podocyte injury. Furthermore, it was revealed that YAP phosphorylation in podocytes was involved in the pathogenesis of diabetic nephropathy by decreasing the expression of transcriptional factor Wilms’ tumor 1 (WT-1) and WT-1-regulated proteins, including nephrin and podocalyxin, all of which are indispensable for podocyte function (6). Also in diabetic nephropathy, the YAP activation of kidney proximal tubule epithelial cells was involved in diabetic tubulointerstitial fibrosis (7). However, it remains a significant knowledge gap regarding how the Hippo pathway engages in the pathogenesis of cGN, despite severe podocyte injury.

## Hippo-YAP overactivation in cGN

The authors commenced by confirming podocyte-specific YAP activation through establishing nephrotoxic serum (NTS)-induced nephritis in mice, a classical model of experimental cGN that is widely used to explore the potential immunological mechanisms of cGN. Increased nuclear YAP expression within podocytes and its downstream targeted genes expression, such as *CCN1* and *CCN2*, were observed in NTS-administered mice, compared with control mice. These gene expression changes preceded crescentic lesions by observation from different time points, indicating that the podocyte Hippo-YAP signaling overactivation is involved in the crescent formation. YAP activity is regulated by its phosphorylation as aforementioned. To further demonstrate that YAP activation within podocytes mediates the pathogenesis of cGN, the authors constructed a mouse model with podocyte-specific YAP activation by mutating all phosphorylation sites of YAP within podocytes (termed P-YAP^5SA^ mice). This mutation led to YAP being constitutively active in the nucleus of podocytes. Unexpectedly, the P-YAP^5SA^ mice without NTS challenge exhibited rapidly deteriorating kidney phenotype over time, mimicking features in human cGN: (i) progressively worsening proteinuria; (ii) cumulative death events due to rapid kidney function loss; (iii) high percentage of cellular crescents and inflammation infiltration. To delineate the underlying mechanisms how YAP activation within podocytes mediates cGN pathogenesis, the authors constructed another mice model of inducible podocyte-YAP activation features relatively mild phenotype which dependent on the extent of YAP activation within podocytes (termed Pi-YAP^5SA^ mice), in contrast to the rapid course of P-YAP^5SA^ mice which hard to capture precise pathophysiological changes. The authors used the Pi-YAP^5SA^ mice revealed two time-sequential events characterized by early extracapillary cell hypertrophy and later cell hyperplasia. Detailed investigation uncovered that the core cells participating in the two events: (i) podocyte hypertrophy and cell-autonomous loss were initial processes to respond to YAP overactivation, (ii) proliferation of activated PECs was a non-cell-autonomous manner to further respond to the podocyte-specific YAP overactivation.

To depict the possible molecular programs involved in the above events, the authors conducted *in vitro* studies by constructing inducible YAP activation in conditionally immortalized and differentiated human podocytes (termed YAP^5SA^ podocytes) and further RNA sequencing analysis. Integrating bioinformatic methods, the authors found several behaviors at the biological and molecular levels in YAP^5SA^ podocytes may be able to explain the phenotype observed in Pi-YAP^5SA^ mice: (i) cytoskeleton and polarity reorganization through reactivation of developmental programs in YAP^5SA^ podocytes corresponds to podocyte injury (foot process effacement) and loss in Pi-YAP^5SA^ mice. (ii) increased expression of GBM-related ECM genes (*Col4a3*, *Col4a4*, *Lama4*, *Lama4*, and *Nid2*) in YAP^5SA^ podocytes corresponds to GBM thickening in Pi-YAP^5SA^ mice. In addition, the authors identified PI3K/Akt/mTOR pathway activation as a downstream mechanism following Hippo-YAP overactivation, leading to podocyte injury. Notably, the proliferation of PECs in a non-cell-autonomous manner, followed by podocyte loss observed in model, indicates a cross-talk between them. The authors further revealed that secretory molecules CCN2 and HB-EGF may be candidates mediating this cross-talk. These “Dry Lab” findings were confirmed by “Wet Lab” experiments in YAP^5SA^ podocytes and their Pi-YAP^5SA^ mice model. To emphasize the translational value of their basic findings, the authors sought to determine the Hippo-YAP signaling activation in cGN patients, including anti-GBM disease, lupus nephritis, and ANCA-associated vasculitis. Expectedly, increased YAP and its downstream target CCN2 expression were observed in podocytes of these patients, but not in patients with non-crescent GN. These clinical findings are aligned with their experimental findings, supporting the role of Hippo pathway-mediated podocyte injury in the pathogenesis of cGN. Interestingly, in the same *issue* of *Science Translational Medicine*, another group from He et al. published a back-to-back original article demonstrating that podocyte-specific YAP/TAZ overactivation, and its upstream signal Last1/2 deficiency, contribute to the pathogenesis of collapsing glomerulopathy (podocyte injury and PECs proliferation are its hallmark features) and ANCA-associated vasculitis (8).

## Conclusion and future direction

cGN is the most severe form of glomerular disease and has the worst prognosis. Despite aggressive nonspecific immunosuppression applied to these patients, most of them eventually struggle to escape lifelong dialysis. The lack of specific therapies is attributed to the poor understanding of its pathogenesis. The study by Turinsky et al., together with He et al., now extend the pathogenesis of cGN towards a novel mechanism by introducing how the Hippo pathway-mediated podocyte injury leads to PECs proliferation and final crescentic lesions in cGN (**Figure 1B**). It also expands our understanding of the role of the Hippo-YAP signaling in podocyte function. More importantly, the Hippo pathway overactivation occurs in all types of cGN, implying that, as an end effector mechanism for cGN occurrence, future therapies targeting this pathway may treat all types of cGN patients regardless of their different upstream etiologies, just like complement inhibition treatment in cGN. Preclinical *in vivo* studies of pharmacological inhibition targeting the Hippo pathway, such as small molecule verteporfin inhibits the YAP-TEAD interaction (9), should be conducted in the future to validate the translational value of the above studies. Finally, as with any revolutionary study, many open questions remain, such as: (i) although quiescent PECs do not appear to show YAP/TAZ overactivation, possibly due to their distance from the center of “immunological hits” in cGN, is it possible that a mechanism existed when proliferation of activated PECs occurs, these PECs also undergo Hippo pathway overactivation thereby further exacerbating crescentic lesions. (ii) In addition to PI3K/Akt/mTOR signaling and candidates CCN2 and HB-EGF, which may mediate podocyte injury and its cross-talk with PECs, respectively, whether other unknown pathways play a role in these critical processes after YAP overactivation in cGN, merits future work. (iii) Synchronization with question (ii), the authors observed complement cascade activation in podocytes of YAP^5SA^, whether this another end effector mechanism in cGN that will interact with YAP/TAZ overactivation and thus participate in its pathogenesis? Because previous studies suggest that the Hippo pathway is regulated by GPCRs, such as the complement receptor C3ar1 (10), which is indeed involved in podocyte injury (11). We anticipate the many innovative advances that this study will spark in the future.
